# A case for community metadata standards in cryo-electron tomography

**DOI:** 10.1042/ETLS20240013

**Published:** 2025-04-29

**Authors:** William Wan

**Affiliations:** Department of Biochemistry and Center for Structural Biology, Vanderbilt University, Nashville, Tennessee, USA

**Keywords:** cryo-electron microscopy, cryo-electron tomography, *in situ*, structural biology, subtomogram averaging

## Abstract

In the past decade, cryo-electron microscopy and single particle analysis (SPA) have quickly become key methods in structural biology. In particular, increased access to equipment and streamlined software has enabled new users to successfully carry out SPA projects. At the same time, cryo-electron tomography (cryo-ET) has also made great technical strides, most notably with cellular cryo-ET. While many challenges remain, developments in hardware and automation have made cellular cryo-ET specimen preparation and data collection more accessible than ever. There is also a growing field of cryo-ET software developers, but the wide variety of biological specimens and scientific goals that can be pursued using cryo-ET makes it difficult to develop processing workflows analogous to those in SPA; this becomes a major barrier to entry for new users. In this perspective, I make a case that the development of standardized metadata can play a key role in reducing such barriers and allow for an ecosystem that enables new users to enter the field while retaining a diversity of processing approaches.

## Introduction

Since the advent of the ‘resolution revolution’ a decade ago [1], cryo-electron microscopy (cryo-EM) has rapidly expanded [2], with single particle analysis (SPA) becoming a standard approach for macromolecular structure determination. This was partly due to the expansion of cryo-EM resources [3], both at individual institutions and, in particular, at large national or multinational facilities, including the National Cryo-EM Centers in the United States, the electron Bio-Imaging Centre at the Diamond Light Source in the United Kingdom, the European Molecular Biology Laboratory imaging center in Germany, as well as transnational consortia such as instruct-ERIC and iNEXT-Discovery. Altogether, these facilities and resources have provided greater access to both instrumentation and expertise to the broader structural biology community. The development of monolithic SPA packages that cover the whole image processing pipeline, most notably RELION [4] and cryoSPARC [5], has also provided easier entry points into data processing. Taken together, the increased access to instrumentation and user-friendly software has enabled new users to successfully perform SPA with no prior cryo-EM experience.

Concurrently, cryo-electron tomography (cryo-ET) and subtomogram averaging (STA) have also experienced a number of technical advances, including improvements in automated data collection [6–8] and high-resolution structure determination [9]. One key development has been the adaptation of focused ion beam (FIB) milling for use with vitrified biological materials [10–12], which has quickly become the standard approach for cryo-sectioning cells. Cellular cryo-ET is unique among structural biology methods, allowing for direct imaging at molecular resolution in near-native environments. As such, cellular cryo-ET not only provides information on individual molecules but, more importantly, also provides information on how these molecules interact and distribute within native systems, i.e. it provides biological context. Access to this type of information makes cellular cryo-ET an emerging tool in the structural biologists’ repertoire, so it is important to consider what the barriers to entry are.

## Current challenges

One key barrier to performing cellular cryo-ET is the higher complexity and lower throughput of specimen preparation and data collection. Adherent cells can be grown directly on EM grids, while non-adherent cells in solution can be applied to grids, similar to purified molecules in SPA [13]. Vitrification can be performed with plunge freezing, though, for thicker cells, high-pressure freezing may be required [13]. After cells are frozen, users may wish to target specific cells or subcellular structures using correlative light and electron microscopy [14]. Finally, cells that are appropriately situated in grid squares can be FIB-milled [11] and their lamella can then be used to collect tilt series data. While much work is left to be done in this area, I would argue that there is already a significant effort to address these issues. There are a number of groups developing FIB-milling approaches for various cell types [15–22], and centers and institutes have been obtaining FIB instruments and providing users with both access and training [3,23]. Automated approaches for both lamella preparation [24–26] and tilt-series collection [6,8,27] are actively being developed with the goal of increasing the ease and throughput of cellular cryo-ET specimen preparation and data collection. Instead, I believe the less appreciated barrier to expanding the usage of cellular cryo-ET is in image processing.

Cryo-ET imaging processing is generally more complex than SPA. For instance, going from raw movies to reconstructed tomograms requires a number of preprocessing tasks not present in SPA [9,28]. After that, the goals in cryo-ET are typically quite varied and may include tasks such as membrane segmentation and morphometrics [29,30], molecular identification [31–33], structure determination by STA [9,28,34], and spatial analyses of molecular species and their subcellular localization [28,35]; each of these require different types of image processing algorithms. Furthermore, image processing in cryo-ET often requires iterative steps through the entire pipeline. In contrast, SPA generally aims to determine structures of biochemical homogeneous specimens using a well-defined set of processing steps. While compositional and conformational heterogeneity are also major concerns in SPA, these are also present, and arguably more complicated, in cellular specimens.

Given the complexity of cryo-ET image processing, it is difficult to develop and maintain monolithic packages that can successfully perform all tasks for all projects. As such, successful cryo-ET projects often depend on the ability of users to develop workflows that best suit their biological systems. This generally requires combining software packages that are developed by different groups with different design philosophies. Chaining the inputs and outputs of these packages together is often difficult and time-consuming for expert users, to say nothing of those trying to enter the field. While the solution may appear to be to push for the development of monolithic packages, I would argue against this, as a diverse ecosystem of packages and algorithms maintains creativity in the field and provides users with a wider set of tools, some of which may be particularly well suited to their specific needs. Packages such as ScipionTomo [36], TomoBEAR [37], and TOMOMAN [38] wrap various external packages and provide other tools to enable users tailor their processing pipelines. However, they each do this with their own internal metadata formats, which still effectively locks users into a specific pipeline, albeit more flexible ones. Instead, the approach I advocate for would be to ease interoperability by defining a standard set of vocabulary and formalisms for common cryo-ET metadata.

## Standardized metadata

Standardizing cryo-ET data and metadata formats can have a variety of benefits, both in and out of the community. For users, standard metadata can minimize the need to convert between different data conventions, such as affine transforms or Euler angles; ideally, only minor scripting may be required. This simplifies moving between software packages, making it easier for users to optimize workflows for their specific biological problems. For developers, improved interoperability reduces the barrier to entry, which can enable more widespread usage of new software packages. For data repositories, standardization can streamline deposition and ensure that all metadata necessary to reproduce published results is present. This is particularly important, as cellular cryo-ET data typically contain much more biological information than can be analyzed by the group that collected it; standardized, well-annotated datasets allow for greater data reuse and collaborative projects while reducing the need to reprocess data. An example of this is the recently deposited *Chlamydomonas reinhardtii* dataset (EMPIAR-11830), which contains 1829 tilt-series [39]. Furthermore, standardization also makes the construction of new resources like cloud visualization tools or AI-based algorithms more efficient, making cryo-ET results more accessible to non-structural biologists.

The implementation of standardized cryo-ET metadata would need to be done in a way that is clear and understandable to users, as well as amenable to the software development community. On the first point, a well-designed standard can be a useful learning tool for new practitioners as it provides precise technical documentation that is not typically covered in the literature. On the second, it is unlikely that there would be a unified standard that developers would build into their image processing algorithms. Instead, the tractable approach would be to develop standard metadata formats that cover all operations required to reproduce the data processing, and for developers to provide tools that read and write into these formats. Given that cryo-ET is still an emerging field, the development of metadata standards should be considered an ongoing process. Basic types of metadata such as affine or rigid-body transformations are unlikely to change, so stronger standards can be implemented. However, more complex information, such as segmentations and morphometrics, are still being developed and would currently benefit from less strict standards. Altogether, standards should be considered living documents that are reassessed and updated as the field progresses. This approach to standardization naturally lends itself to data archival, and stakeholders such as Electron Microscopy Public Image Archive (EMPIAR) [40] and the Chan Zuckerberg Imaging Institute have already convened a working group of software developers to draft an initial set of standards. Additionally, a consortia of the Swiss cryo-EM facilities are developing metadata standards for data collected at their facilities under the OpenEM project [41].

## Conclusion

The biggest issue in implementing metadata standards is ensuring that they are accepted by the community. Historically, the adoption of standards has been mixed, with some, such as EMX (electron microscopy eXchange), failing despite contributions from prominent developers [42]. To a significant degree, fragmentation and difficulties in adoption is to be expected as a new field develops and expands. However, successful standards have been developed through collaborations between developers and repositories such as the Protein Data Bank (PDB) or Electron Microscopy Data Bank (EMDB), which are a part of the world-wide PDB organization [43–45], arguably due in part to the unique ability of repositories to enforce standards for deposition. While I think it may be up to EMPIAR to play such a role [46], such enforcement only applies to the few instances where raw data are deposited, making it easy for most to view this as a low priority. As such, the greater problem is arguably in convincing the community of the importance of data sharing. While funders can play a role in this, e.g. the National Institute of Health’s recently implemented data sharing policies that mandate the deposition of data at the end of funding periods, I think it is more important that this becomes an norm within the community. To this end, I am optimistic about the future, as the new generation of researchers is intimately familiar with the effort required to produce and process cellular cryo-ET data; most of them want to ensure that their work has a wide impact on the community beyond the initial publications.

## Abbreviations

EMDB: Electron Micoscopy Data Bank
EMPIAR: Electron Microscopy Public Image Archive
EMX: Electron Microscopy eXchange
FIB: focused ion beam
PDB: Protein Data Bank
SPA: single particle analysis
STA: subtomogram averaging
cryo-EM: cryo-electron microscopy
cryo-ET: cryo-electron microscopy
